# Tellurium: A Rare Element with Influence on Prokaryotic and Eukaryotic Biological Systems

**DOI:** 10.3390/ijms22115924

**Published:** 2021-05-31

**Authors:** Silvia Vávrová, Eva Struhárňanská, Ján Turňa, Stanislav Stuchlík

**Affiliations:** 1 Department of Molecular Biology, Faculty of Natural Sciences, Comenius University, Ilkovičova 6, 842 15 Bratislava, Slovakia; siliva.vavrova@uniba.sk (S.V.); struharnans1@uniba.sk (E.S.); jan.turna@uniba.sk (J.T.); 2 Science Park, Comenius University, Ilkovičova 8, 841 04 Bratislava, Slovakia

**Keywords:** tellurium, tellurite resistance, human diseases, oxidative stress

## Abstract

Metalloid tellurium is characterized as a chemical element belonging to the chalcogen group without known biological function. However, its compounds, especially the oxyanions, exert numerous negative effects on both prokaryotic and eukaryotic organisms. Recent evidence suggests that increasing environmental pollution with tellurium has a causal link to autoimmune, neurodegenerative and oncological diseases. In this review, we provide an overview about the current knowledge on the mechanisms of tellurium compounds’ toxicity in bacteria and humans and we summarise the various ways organisms cope and detoxify these compounds. Over the last decades, several gene clusters conferring resistance to tellurium compounds have been identified in a variety of bacterial species and strains. These genetic determinants exhibit great genetic and functional diversity. Besides the existence of specific resistance mechanisms, tellurium and its toxic compounds interact with molecular systems, mediating general detoxification and mitigation of oxidative stress. We also discuss the similarity of tellurium and selenium biochemistry and the impact of their compounds on humans.

## 1. Tellurium and Its Occurrence

Tellurium (Te) is a member of the chalcogen group, which includes oxygen, sulphur, selenium (Se) and polonium [1]. The first three members of the chalcogen group have crucial functions in biochemistry, biology and medicine, whereas Te is a strange element with no apparent role in biological systems. Moreover, it belongs to the group of very few elements in the Periodic Table that have been almost completely ignored. Elemental tellurium Te^0^ has the chemical properties of a nonmetal or metalloid and the physical properties of a metal. Its chemistry is similar to that of Se in many ways [2]. There are many not yet fully understood mechanisms of Te compounds in organisms but Se, with many similarities in properties, can provide a considerable example.

Te is a rare element of the Earth’s crust, with a low global and heterogeneous distribution and natural abundance of only about 10^−2^ to 10^−8^ ppm [1,3]. Information on environmental concentrations of Te is scarce and often misleading due to inadequate analytical methods. Recent known values in surface water indicate concentrations of a few to low tens of ng/kg [4,5]. There are unknown data about elemental Te concentrations in crust [5], and values for soil concentrations differ with a broad range from 0.008 mg/kg to 0.03 mg/kg [5], and in some localities that are naturally anthropologically enriched with Te, compounds can reach from 0.166 mg/kg [6] even up to 0.5 mg/kg [7]. Because of human industrial activities, Te-containing compounds can arise as environmental pollutants [8,9]. Te compounds are mostly found as tellurides of copper, gold and silver ores [10]. In areas adjacent to gold mines, the concentration of Te reaches extreme values of up to 14.8 ppm [11]. Te is also a by-product of the electrolytic refining of copper. During this process, the anode muds produced as a by-product are commercially used as they contain up to 8% Te. Te forms alloys with other metals, mainly copper, gold and silver [12], and can be incorporated into stainless steel, lead and bronze to improve their machinability and to make them more resistant to corrosion. Te has been used to create photovoltaic modules [13] and high-efficiency thermoelectric generators [14], in the vulcanization of rubber (in which it increases resistance to heat, abrasion and aging) and in metal-oxidizing solutions to blacken or tarnish metals (in jewellery manufacturing) [15]. Tellurite can form part of the pigments for the colouring of glass and ceramics [16], which is present in rechargeable batteries [17] and is also a component of solar panels and rewritable CDs and DVDs. Its occurrence in places with high anthropogenic industrial activities [18] causes environmental problems that not only destroy the microbial ecology of soil but also pose a serious risk to human health, since Te compounds are highly toxic with a teratogenic effect in rats [19]. The elementary Te^0^ has been classified as non-toxic for living forms [20] in comparison with soluble Te oxyanions [21].

Te compounds can be divided into three groups: (a) inorganic tellurides; (b) Te-containing complex-like structures; and (c) organotellurides [2]. Inorganic Te compounds occur be in various oxidation states ranging from -II to +VI, namely -II (H_2_Te, hydrogen telluride), 0 (Te^0^ elemental tellurium), +II (TeO, tellurium monoxide), +IV (TeO_3_^2−^, tellurite) and +VI (TeO_4_^2−^ tellurate). These oxides form tellurous acid (H_2_TeO_3_) and telluric acid (H_2_TeO_4_) and their salts are known as tellurites TeO_3_^2−^ and tellurates TeO_4_^2−^. The anion TeO_2_^2−^ in the oxidation state of +II also exist [1,22]. Te-containing complex-like structures have been studied extensively in a biological context. They contain a central Te atom, which is bound to a range of ligands. Ligand changes are responsible for the majority of the biological activities of various cysteine proteases [23]. For instance, an active site change at the Te atom results in the inactivation of cysteine protease. This process has a wide range of implications in connection with tumour invasion. Organic Te agents have GPx-like activity, as shown by tests in vitro in which organotellurides have been used as mimics of the antioxidant GPx (glutathione peroxidase), because of their resemblance to the human Se-containing enzyme GPx [24,25]. Some organotellurides have a biochemical activity that mimics vitamin E [26].

## 2. Effects of Tellurium and Its Compounds on Selected Prokaryotic Systems

### 2.1. Mechanism of Toxicity

Tellurites are highly toxic, even at micromolar levels (1 µg/mL) [27,28,29]; however, there are no recorded thresholds according to the OSHA and NIOSH databases. The toxicity of potassium tellurite (K_2_TeO_3_) was recorded for the first time by Sir Alexander Fleming in 1932 [30]. These compounds were used for the treatment of some medical conditions such as syphilis, tuberculosis, cystitis, dermatitis, eye infections and leprosy. Therefore, Te-containing soluble salts were historically used as antimicrobial and therapeutical agents before the era of antibiotics [1].

Te probably exploits the metabolic machinery of Se, and tellurodiglutathione (GSTeSG), which is an analogue of selenodiglutathione (GSSeSG), is produced during H_2_Te formation. The toxicity mechanism of thiol-binding metal(loid)s is based on the interaction and subsequent inhibition of essential thiol groups of enzymes and proteins [31]. The similar physical and electrochemical features of Te to Se and sulphur lead to its substitution of them in proteins [32]. The erroneous incorporation of the resulting tellurocysteine and/or telluromethionine into protein structure leads to changes in protein activity or protein inactivity.

Upon the entry of TeO_3_^2−^ into a cell, the transmembrane proton gradient is disrupted in *E. coli*, irrespective of the level of resistance. This effect is accompanied by the inhibition of ATP synthesis, resulting in the depletion of intracellular ATP stores during aerobic growth [33]. The same detrimental effect of Te compounds has been found in protein synthesis with regard to proteins containing amino acids with reduced thiol groups [34] of both low-level and high-level resistant microbes. Two high-resistant bacteria, namely *Erythromonas ursincola* strain KR99 and *Erythromicrobium ramosum* E5, are distinct from other microbes, both showing an increase in protein and ATP synthesis in the presence of Te compounds [35]. The mechanism of this increase is uncertain, but it was implied [35,36,37,38] that reduction of such oxyanions could help with retaining optimal redox balance.

The reduction of Te compounds in modified cell culture media is accompanied by black colony formation. This chromogenic-based selective cultivation has been used for the diagnosis of the presence of antibiotic-resistant pathogenic bacteria for decades and, despite the emergence of genomic approaches, remains widely used [39,40,41].

### 2.2. Mechanism of Tellurite Resistance

Three main types of resistance exist: intrinsic, acquired and adaptive.

Intrinsic resistance comprises the inherent properties of a microorganism that limit the action of antimicrobials. The mechanisms of bacterial resistance to toxicants, in general, are extraordinarily diverse. They can be specific, whereby the main role of the cell is to resist the action of a toxic compound, or nonspecific, in which the resistance is based on a component that is involved in other cellular functions but that also exerts a protective effect against the toxicant. The nonspecific mechanisms are carried out at low levels of resistance/reduction [42] and can be enzymatic or non-enzymatic. Resistance can be achieved by mutations that affect the intracellular target for a given antimicrobial drug. Even if the toxicant enters the intracellular milieu and target affinity does not change, bacteria can enhance their resistance by actively expelling the toxicant from their cells by efflux. Another mechanism is based on the reduced entry of the toxicant into the bacterial cell resulting from modifications to the cell surface that restrict interactions with the toxicant or from a reduction in the number of entry channels, such as porins. In the case of no permeability changes, the generally reduced uptake of toxicant through the semipermeable outer membrane acts in synergy with other resistance mechanisms such as enzymatic degradation and efflux [43], for instance, the possession of a semipermeable outer membrane with low permeability, as is the case for the Gram-negative pathogens *Pseudomonas aeruginosa* and *Acinetobacter baumannii*, or the constitutive efflux pumps observed in many bacteria.

Acquired resistance occurs when an originally sensitive microbe becomes resistant by incorporating new genetic material (e.g., plasmids, transposons, integrons and naked DNA), by lateral gene transfer, or as a result of mutation. These mutational events may lead to a large increase in the minimal inhibitory concentration [43,44,45]. 

Adaptive resistance involves a temporary increase in the ability of a bacterium to survive in the presence of a toxicant by alterations in gene and/or protein expression as a result of exposure to an environmental trigger, e.g., stress, nutrient conditions, growth state and sub-inhibitory levels of the toxicant or antibiotics themselves.

Intrinsic and acquired resistance mechanisms are stable and can be transmitted vertically to subsequent generations. Adaptive resistance has a transient character and usually reverts upon the removal of the inducing condition [43]. 

Several microorganisms that demonstrate tolerance/resistance to Te compounds have been isolated from various environments [46]. They are able to cope with the toxic environment of TeO_3_^2−^ in diverse ways. The first group of organisms horizontally obtained genetic equipment harboured in mobile genetic elements, the most commonly being plasmids, or integrated such genes (e.g., *ter* genes in *E. coli*) into their chromosomes and organized them into genomic islands consisting of gene clusters or operons with various gene compositions [47]. Similar gene compositions within a genomic island indicate a lateral transfer from a common ancestor, anciently predestined for survival in the metal-rich environment [42,48]. The second group of organisms evolved other mechanisms enabling cellular enzymes to be used for the reduction of the noxious effects of toxicants. In the first-mentioned group, gene clusters enable resistance against toxicants, whereas the latter triggers the ability to tolerate/adapt to life in a toxic environment.

#### 2.2.1. Resistance via the Modulation of Tellurite Efflux

**Figure 1:**
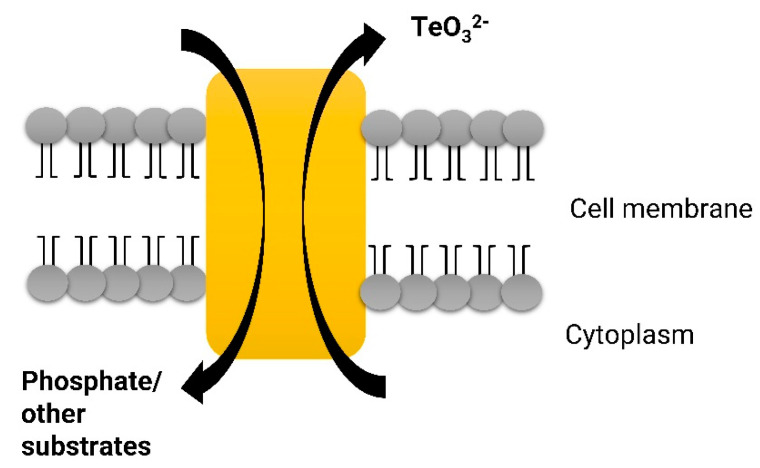
Schematic pathway of efflux of TeO_3_^2−^.

Tomas and Kay [49] suggested one mechanism of TeO_3_^2−^ resistance. They demonstrated that TeO_3_^2−^ is transported via the phosphate transport pathway in *E. coli* [50,51] (Figure 1). The mechanism of such a type of resistance is based on either a chromosomal mutation or on the acquisition of a TeO_3_^2−^ resistance plasmid that leads to the inability to transport TeO_3_^2−^, the acquisition of an efflux mechanism or the detoxification of the inhibitor [51]. The direct efflux mechanism evolved to prevent the intracellular accumulation of toxic compounds and involves energy-dependent systems that pump such molecules out of the cell in a process that does not alter or degrade the toxicants [43]. However, the direct efflux of TeO_3_^2−^ does not constitute a specific resistance mechanism. A decreased influx and increased efflux of TeO_3_^2−^ is not responsible for the TeO_3_^2−^ resistance of *E. coli* [52]. Subsequent observations by Borghese et al. [53] revealed that a decreased uptake by an acetate transport system, based on tellurite-acetate competition, is responsible for TeO_3_^2−^ uptake and resistance in *Rhodobacter capsulatus* [54].

#### 2.2.2. Resistance via Reduction of Tellurite

One of the mechanisms of TeO_3_^2−^ toxicity is based on its high oxidizing ability. The selection of resistance to TeO_3_^2−^ and of the ability of some bacteria to reduce TeO_3_^2−^ to less toxic elemental Te has been carried out for decades in specific media containing TeO_3_^2−^ in various concentrations. As mentioned above, the reduction of Te/Se compounds in such modified cell media is accompanied by black/red colony formation, respectively. Despite the emergence of genomic approaches, this chromogenic-based selection continues to be extensively used [39,40].

Several key cellular enzymes are reported to be involved in the defence against ROS generated during TeO_3_^2−^ reduction [55]. The first reports describing bacterial colonies with a black phenotype were published by Klett [56] and Scheurlen [57]. These authors described the production of black or grey insoluble Te^0^ in microorganisms treated with TeO_3_^2−^. This phenomenon is caused by the reduction of TeO_3_^2−^or Te^4+^ to the less toxic Te^0^. Regardless of the concentration of TeO_3_^2−^, this results in the generation of black or grey deposits of metallic Te^0^ inside the cell [20]. The same observation was made by Yurkov et al. [58] who also pointed out that TeO_3_^2−^ reduction accompanied by black/grey colony formation and metallic Te crystal formation in obligate aerobic photosynthetic bacteria was not essential for the maintenance of high TeO_3_^2−^ resistance. White-colony formation without TeO_3_^2−^ reduction has also been described in highly tellurite-resistant *E. coli* strains [59]. Many non-tellurite resistant microorganisms are able to reduce TeO_3_^2−^ at low concentrations, one such example being *E. coli* K12 [27,60]. A major mechanism of resistance involves the inactivation of the toxicant by enzymes. The reduction of TeO_3_^2−^ to Te^0^, in this case, can be also carried out by the activity of the plasma membrane flavin-dependent reductase [38,61]. Several types of non-specific/housekeeping enzymes can be involved in the detoxification process of TeO_3_^2−^ reduction, e.g., the thiol:disulphide oxidoreductase of *Rhodobacter capsulatus* [62] and GutS of *E. coli* [63]. Catalases are also involved in the detoxification of TeO_3_^2−^ by affecting its reduction state in *Staphylococcus epidermidis* [64]. In addition, periplasmic and membrane-associated nitrate reductases have been described in *E. coli* and *Rhodobacter sphaeroides*; although having a diverse primary function, all of these non-specific enzymes are known to confer low-level TeO_3_^2−^ resistance [60,65]. Details of only three specific TeO_3_^2−^ reductases have been published to date: the cytoplasm-localized reductase of *Bacillus* spp. [66,67], the periplasmic TeO_3_^2−^ reductase of the Gram-negative facultative anaerobe *Shewanella fridigimarina* [68] and the membrane-associated reductase of an aerobic anoxygenic phototroph *Erythromonas ursincola* [69].

#### 2.2.3. Resistance via Formation of Tellurium Organic and Volatile Compounds

Bonificio and Clarke [70] have proposed a steady-state system of Te compounds in *Pseudoalteromonas* spp. strain EPR3. They have demonstrated that, even though many Te compounds are considered insoluble, they can nonetheless be transformed and suggest that a steady-state soluble Te concentration exists during Te transformation. This system is based on solid Te sources (tellurium dioxide, autoclave slime, cadmium telluride, bismuth telluride) being able to dissolve to yield soluble TeO_3_^2−^. The TeO_3_^2−^ enters the cell and the bacterium transforms it to either metallic elemental Te or a gaseous Te species (dimethyl telluride C_2_H_6_Te) [20,71,72]. The system stays in a steady-state in which the undissolved Te source, the metallic Te, TeO_3_^2−^ and volatile Te compounds are present. Solid elemental Te dissolves to TeO_3_^2−^, which can be converted to a gaseous Te species and subsequently excreted to the environment by volatilization [73]. Volatile Te species might also be transformed back to TeO_3_^2−^ [70]. The production of organic Te compounds, such as dimethyltelluride, is considered to be one of the detoxification mechanisms of bacteria [3,72]. 

#### 2.2.4. Ter-Gene-Mediated Resistance

Jobling and Ritchie [74] described, for the first time, a TeO_3_^2−^ resistance (ter) determinant of the plasmid pMER610 of *Alcaligenes* sp. Subsequently, plasmid pR478 harbouring additional *ter* genes was found in the opportunistic pathogen *Serratia marcescens* [75]. The nosocomial pathogen *Klebsiella pneumoniae* CG43 possesses the *ter-*gene-containing plasmid pLVPK [76]. Several different *Escherichia coli* pathogens also possess *ter* gene clusters. The uropathogenic *E. coli* KL53 strain [59,77,78] and the widely known foodborne pathogen *E. coli* O157:H7 [79,80] have *ter* gene clusters incorporated into large plasmids. *Ter* genes have also been identified as part of the chromosomal genomic island of *E. coli* O157:H7, an interesting observation in the context of horizontally transferred pathogenicity islands as tools of microbial evolution [47,81]. The other described TeO_3_^2−^ resistant strains are *Vibrio cholerae* [49], *Corynebacterium diphtheriae, Staphylococcus aureus, Shigella* spp. [82], the TeO_3_^2−^- and SeO_3_^2−^-resistant bacteria of hydrothermal vents *Pseudoalteromonas* sp. [83], *Bacillus subtilis* and *Yersinia pestis* [84]. Muñoz-Villagrán et al. [85] have described a new TeO_3_^2−^-resistant Antarctic strain, namely *Psychrobacter glacincola*, which has great biotechnological potential for low-temperature applications and bioremediation. The most recent strain showing great promise in the bioremediation of toxic Te(IV) contamination is *Shinella* sp. [86]. Furthermore, a TeO_3_^2−^ resistance protein, namely AtTerC, has been detected that is also a thylakoid membrane protein involved in the assembly of photosystem II (PSII) in *Arabidopsis thaliana*. TerC seems to be a crucial protein, as its knockdown leads to the seedling-lethal phenotype [87]. TerC is a member of the YidC/Oxa1/Alb3 family. The homologues of this protein family are ubiquitously distributed in organisms. They function in bacteria, mitochondria and chloroplasts as membrane protein insertases. All homologues are involved in the insertion, folding and assembly of membrane proteins [88]. Predicted homologues of TerC are present in many bacteria, in plants and green algae and in some but not all cyanobacteria [89].

A summary of all TeO_3_^2−^ resistance mechanisms proposed to date is provided in Table 1. However, some of the mechanisms are not as yet supported by experimental evidence.

## 3. Tellurium Toxicity vs Potential Benefits for Prokaryotes and Eukaryotes

### 3.1. Impact of Tellurium Compounds on Organisms

When Te compounds enter into cells, they can induce (I) changes in the integrity of cellular membrane structures [90], (II) changes in glutathione metabolism, (III) substitution of metal in enzymes and (IV) oxidative stress [91]. The common feature of these metal(loid)s is their chemical affinity to proteins and to non-protein thiols and their ability to generate cellular oxidative stress by the Fenton reaction. Oxidative stress is induced by their interaction with the cell thiolome [92,93], which represents the entity of the cellular thiol pool. Molecules carrying thiol groups are mostly derivatives of the amino acid cysteine and are called low molecular weight (LMW)-thiols: coenzyme A (CoA), glutathione (GSH) or bacillithiol (BSH). They serve to balance a reduced cell environment, act as cofactors in enzymatic reactions or help in the detoxification of reactive oxygen or nitrogen species, electrophilic compounds, or thiophilic metalloids (AsO_3_^2−^, TeO_3_^2−^). Te oxyanions (TeO_3_^2−^) are involved in the thiol: redox system of the cell and interfere with thiol: redox enzymes (glutathione reductase and thioredoxin reductase) and with their metabolites (glutathione, glutaredoxin and thioredoxin) [52]. The key target for SeO_3_^2−^ and TeO_3_^2−^ cellular processing is glutathione, which participates in TeO_3_^2−^ to Te (Te^0^) reduction [94] accompanied by reactive oxygen species (ROS) formation [95]. 

Glutathione is an endogenous tripeptide consisting of cysteine, glutamate and glycine and has antioxidative and other metabolic functions [96]. Glutathione and sodium glutathione are used to prevent neurotoxicity associated with cisplatin or oxaliplatin during cancer treatment and can also prevent adverse effects of antineoplastic and radiation therapy. They can additionally be used in the treatment of a wide range of other disorders including poisoning with heavy metals and other compounds and even in COVID-19 disease therapy [97]. The biosynthesis and metabolism of glutathione are directly related to stress tolerance. Noctor et al. [98] have demonstrated, in their review, that the metabolic pathway of glutathione biosynthesis consists of two sequential ATP-dependent reactions allowing the synthesis of γ-glutamylcysteine from L-glutamate and L-cysteine, followed by the formation of glutathione by the addition of glycine to the C-terminal end of γ-glutamylcysteine [99]. These reactions are catalysed by γ-glutamylcysteine synthetase and glutathione synthetase.

### 3.2. Impact of Tellurium and Selenium on Humans

The metabolic pathways involving Te, the mechanisms of its toxicity and its impact on human health, have been studied poorly to date. Te compounds can be taken in by inhalation or ingestion but neither pathway is often described. The clinical manifestation of the ingestion of metal-oxidizing solutions containing substantial concentrations of Te include vomiting, nausea, metallic taste, black discoloration of the oral mucosa and skin, corrosive gastrointestinal tract injury and a characteristic garlic-like odour of the breath [15]. As mentioned above, organotellurium compounds are, in general, less toxic than inorganic Te compounds. They have in part different pharmacological and pharmacokinetic profiles from each other, and they overcome different metabolic conversions in the human body. A few publications mention Te toxicity connected with microorganisms [20] and, thus, a possible bridge between microbial and human cells can reasonably be considered. One experimental observation is the analogy in the discoloration of skin versus that of bacterial cells. Phenotypic changes attributable to the reduction of TeO_3_^2−^ to elemental Te (Te^0^) are accompanied by the blackening of colonies and/or media as a consequence of the presence of nanocrystals or nanoparticle formation in the periplasmic space of cells [100]. Yarema and Curry [15] suggest that the phenomenon of discoloration of the skin is a result of the deposition of elemental Te in the dermis and subcutaneous tissue. Exposure to gaseous hydrogen telluride is different from exposure to other forms of Te. When exposure is limited, the mucous membrane and pulmonary system might become irritated. In animals, extensive exposure to hydrogen telluride have resulted in haemolysis, haemoglobinuria, anuria, jaundice and pulmonary oedema, symptoms similar to toxicity from the inhalation of arsine or the poisonous gas stibine [101]. The toxicity of Te, Se and As resides is associated with analogical secondary metabolite production, e.g., dimethyl telluride ((CH_3_)_2_Te), dimethyl selenide ((CH_3_)_2_Se) and monomethyl (CH_3_AsO(OH)_2_) and dimethyl arsenic acid ((CH_3_)_2_AsO_2_H), all of which have a characteristic garlic odour. Their toxicity is exerted via strong interaction with cysteine-containing proteins and enzymes [2]. 

The activity of many more enzymes is affected by Te compounds in animals. Experiments on rats and mice have revealed that, after TeO_3_^2−^ ingestion, the peripheral nerves become transiently demyelinated because of the inhibition of squalene epoxidase (squalene monooxygenase) [102]. This enzyme is involved in the biosynthesis of cholesterol, which is a crucial component of myelin. The mechanism of squalene epoxidase inhibition resides in the binding of methyltellurium compounds or TeO_3_^2−^ itself to the thiol groups of the cysteine residues at the active site [103]. Te atoms attack sulphur-containing cysteine-proteases, such as cathepsin B and caspases, which can lead to changes in cellular metabolism resulting in cell death via apoptosis. The same consequences can be observed after selenium-tellurium interaction in less abundant selenocysteine-containing biomolecules, such as selenoprotein P, thioredoxin reductase (TrxR) and various GPx enzymes [104]. Inhibition of these enzymes by interaction with Te compounds triggers a loss of antioxidant defence and widespread oxidative stress. Another group of enzymes affected by organotellurium compounds toxicity belongs to the glutamatergic system. Neurotoxic diphenyl ditelluride is able to alter enzymes of the glutamatergic system because of its interaction with the thiol groups of cysteine-containing enzymes and proteins [105]. The abovementioned group of enzymes use tellurium-sulphur chemistry to exert the toxicity of Te compounds. Tellurium-selenium interaction also has a significant effect on the activity of enzymes involved in Te toxicity. One member of this group is the human selenoenzyme thioredoxin reductase (TrxR), whose activity is modified by tellurium-selenium interaction [106]. These tellurium-sulphur and/or tellurium-selenium bonds can equally change the protein function and abolish normal enzymatic activity and can cause extensive damage to the cell via oxidative stress leading to cell death. Both Te compounds and Se compounds (I) weaken the cell´s antioxidant defence (II) actively generating ROS. 

The human body is known to metabolize and excrete Te, even if the exact metabolic pathways are not understood. However, a parallelism with the pathways of Se is apparent. After ingestion of TeO_3_^2−^ and TeO_4_^2−^, both are reduced in the liver. TeO_3_^2−^ is methylated resulting in dimethyltellurium ((CH_3_)_2_Te) and finally trimethyltellurium ((CH_3_)_3_Te^+^). Dimethyltellurium can bind to haemoglobin, which then accumulates in the blood cells of the rat. This interaction resembles the action of arsenic [107]. Ba et al. [2] suggest that these methylated species are the most abundant forms of Te in the human body; they are found in the kidney, in the spleen and in the lungs. Finally, Te leaves the human body via urine and via breath as volatile (CH_3_)_2_Te, which is responsible for the garlic-like odour.

#### Chemical Similarity of Tellurium to Selenium Determines Its Biology

The chemical elements Te and Se are members of the chalcogen group, with Se forming part of the amino acid selenocysteine. The selenocysteine-containing biomolecules, are essential for the cell to resist oxidative stress condition [108]. Hence, Se is an essential trace element for both prokaryotic and eukaryotic biological systems at low concentrations but is toxic at higher levels [109]. 

The importance of naturally present trace elements in human food is undisputed. Mineral trace elements such as Se, zinc and copper are members of the group of micronutrients with antioxidative ability. Organic Se in food is found in selenomethionine and selenocysteine, whereas inorganic Se occurs as SeO_3_^2−^ (more toxic form) and selenate (SeO_4_^2−^, less toxic form). These inorganic forms are mobile, toxic and water-soluble. The bioavailability of these compounds allows them to be easily absorbed by plants and animals from Se-rich soil or water. Thus, Se can enter the food chain and poses a potential threat to animals and humans [110,111].

High toxicities of Se and Te oxyanions cause environmental problems in contaminated soils and waters [112]. The ability of microorganisms to reduce Te and Se compounds in polluted and industrial areas is greatly appreciated in biometallurgy and bioremediation. These biotic methods involving the microbial retrieval of elements have become increasingly popular. Many bacterial strains have been described to be able to reduce these toxic oxyanions by producing elemental Se^0^/Te^0^ and by their ability to form nanoparticles containing Se/Te as a result of detoxification. These nanoparticles have various shapes (nanospheres, nanorods, nanowires and nanotubes) [113], sizes and localisations (extracellular, intracellular). *Duganella violacienigra* can reduce both Se and Te oxyanions and so it can be exploited in bioremediation and in eco-friendly approaches to produce rare element nanoparticles, rather than synthesising them by chemical means [29]. Se, Te and other elements such as cadmium (Cd) and sulphur (S) are also used in the production of semiconductor nanoparticles (NPs) or quantum dots (QDs) with unique fluorescent properties and great technological potential [114,115]. *Shewanella oneidensis* MR-1 is another metal-reducing bacterium that reduces TeO_3_^2−^ giving intracellularly accumulated needle-shaped crystalline Te^0^ nanorods. These metal-reducing bacteria, in general, play an important role in the recycling of toxic Te elements and can be applied as a novel selective biological filter for the cellular accumulation of industry-applicable rare elements such as Te^0^ nanorods [90]. Te-containing nanoparticles also exhibit antibacterial properties against *E. coli*, with no apparent cytotoxicity against eukaryotic cells [116]. These nanoparticles provide a huge field for research with potential applications in medicine, pharmacy, optic, metallurgy, chemistry and electronics. Not only are bacteria able to reduce Te, but other organisms such as plants or fungi are also able to accumulate toxic Te elements that can be applied as novel selective biological filters for use in bioremediation processes [90]. SeO_3_^2−^/TeO_3_^2−^ chemical similarity indicates they probably use the same pathway for the accumulation of Se in plants. The latter can proceed via several mechanisms, including phytoremediation, rhizofiltration, phytodegradation, phytostabilisation and phytovolatisation [117]. In connection with Te, plant accumulation from the soil is preferred and the further harvesting and absorption of Te from polluted water via plant roots for further decontamination holds promise [118]. Certain fungi can process Te compounds by biomethylation leading to the production of volatile dimethyltelluride (CH_3_)_2_Te, which is removed from organisms via evaporation [71]. The NaTeO_3_ presented in fungi medium without a sulphur source affects Te-containing amino acid production and consequently the formation of Te-containing protein with modified redox behaviour and catalytic activities [2].

Microorganisms, in general, play a crucial role in the biological transformation of SeO_3_^2−^ and SeO_4_^2−^ via metabolic reactions. The reduction of both forms, SeO_3_^2−^ and SeO_4_^2−^, to Se^0^ has been identified as an ideal strategy for Se detoxification and Se recovery in contaminated water, soil and industrial effluent [119]. A variety of microorganisms are used as microbial factories for the bioproduction of Se nanoparticles, e.g., *Enterobacter cloacae* [120], *Bacillus cereus* [121], *Duganella* sp., *Agrobacterium* sp. [122], *Bacillus mycoides* [123], *Shewanella oneidensis* [124], *Pseudomonas putida* [125] and *Vibrio natriegens* [126].

The concentration of Se in food depends on environmental conditions and thus its concentration in soil [127]. Bacteria can convert an inert form of elemental Se to soluble SeO_3_^2−^ and/or SeO_4_^2−^. The food intake of nutrient antioxidants is therefore considerably reduced in comparison with recommendations in Europe. The best way to supplement food lacking antioxidants is their addition in an inorganic form or rather as selenoproteins. The yeasts *Saccharomyces cerevisiae* and bacterial strains *Escherichia coli* and *Lactobacillus* spp. can accumulate, metabolise and convert SeO_3_^2−^and SeO_4_^2−^ into Se and incorporate it into cysteine and/or methionine [128]. The presence of Se^0^ in yeast cell structures has also been reported by Jiménez-Lamana et al. [129]. The various bacterial strains can also reduce and deposit metal(loid)s, e.g., Te and/or Se in a crystalline form [59]. Some acetic acid bacteria can produce an amorphous metabolizable form of Se or incorporate it into metalloproteins [130,131,132]. 

Since the chemistry of Te slightly resembles that of sulphur, Te can be incorporated into amino acids such as cysteine and methionine and subsequently into proteins and enzymes, as mentioned above. This incorporation does not require special machinery. It can occur naturally via bioincorporation [133]. Telluromethionine and tellurocysteine are suitable for highly sensitive fluorescent imaging methods as biomarkers. Te and its radioactive isotopes ^123m^Te can be synthesized as compounds with fatty acids. They are hardly metabolised but can be incorporated into the myocardium of experimental animals [134]. Te-containing fatty acids, Te-amino acids and many fluorescent Te particles (e.g., CdTe, CdSeTe, CdHgTe and CdTe-ZnTe) and nanoparticles are used as biomarkers for these imaging methods and could have potential applications as nanosensors [113].

Compared with Se-containing compounds (e.g., ebselen and selenocystamine), some of the tellurides are significantly effective compounds. Some of the organotellurium compounds exhibit promising antioxidant activity in cell culture. For example, 4,4′-dihydroxydiphenyltelluride has turned out to be more protective in an (OS)-related model of Alzheimer´s disease [135] in comparison with the Se-containing antioxidant ebselen, which is a synthetic organoselenium drug molecule with anti-inflammatory, antioxidant and cytoprotective activity. It acts as a mimic of glutathione peroxidase and can also react with peroxynitrite (ONOO^−^) [136]. Preliminary studies have demonstrated that ebselen exerts promising inhibitory activity against COVID-19 in cell-based assays. The effect has been attributed to irreversible inhibition of the Coronavirus Main Proteinase (3CL^pro^) via covalent bond formation with the thiol group of the cysteine (Cys-145) of the active centre [137]. The ability of water-soluble organotellurium compounds to catalyse the destruction of zinc-sulphur clusters in vivo has also been tested. Jacob et al. [135] suggest that these compounds might lead to the development of a new class of water-soluble Te-based antioxidant and Zn-releasing drugs. Synthetic organotellurium compounds such as bis(4-aminophenyl) telluride have potent antioxidative properties; bis(4-aminophenyl) telluride has higher glutathione peroxidase-like activity and protects against peroxynitrite-mediated oxidation more efficiently than its Se analogue or ebselen [138]. These organotellurium compounds have potential therapeutic properties as antioxidants and as regulators of Zn metabolism [135].

Inorganic salts of Te and a wide range of diverse organotellurium compounds show potential in diagnostics, pharmacology and therapy. They may provide the basis for innovative drug development in the future [2]. They are powerful agents in protein and enzyme inhibition, they can kill a wide range of microorganisms including bacteria and plasmodia and they are able to induce apoptosis of specific cancer cells.

Extreme environments are rarely distributed evenly over the Earth but they harbour a relatively high proportion of microorganisms considered valuable to science and technology [139], such as bacteria producing antibiotics [140] and bioactive molecules and bacteria useful in the biodegradation of pollutants [141]. Such environments provide habitats for representatives of various genera that possess the ability to resist and reduce elevated levels of toxic metal(loid)s compounds, specifically, Te compounds. These extreme environments are rich in heavy metals and metalloids and present excellent sites for the isolation of metal-resistant microorganisms.

## 4. Conclusions and Perspectives

Tellurium is a rare element with no apparent role in biological systems. Despite considerable advancement in the clarification of the biochemistry of tellurite metabolism tellurium function in biological systems as well as the molecular basis of tellurite toxicity, universal biomolecular mechanism of biochemical detoxification, mechanism of resistance and its interference with cellular processes, have not been elucidated to date and thus selenium can pass as a model.

Toxic chemicals, such as heavy metals or toxic compounds of some elements, can contribute to changes in human gut microbiota composition and metabolic profile due their uptake. It is important to understand in future works how these chemicals affect gut microbiota in relationship with metabolic and environmental diseases.

## Figures and Tables

**Table 1:** Proposed putative tellurite (TeO_3_^2−^) resistance mechanisms.

Description of Considered Mechanism of Resistance	Organism	Reference
Inability to transport TeO_3_^2−^ into the cell because of competitive inhibition of TeO_3_^2−^ by phosphate. TeO_3_^2−^ is transported by phosphate transport pathway.	*Escherichia coli*	[57]
Oxidation-reduction steady-state mechanism among solid, soluble and volatile gaseous Te species. TeO_3_^2−^ conversion to volatile organic Te compounds.	*Rhodotorula* spp. *Pseudoalteromonas* spp. *Bacillus* spp.	[3] [76] [77]
Direct efflux of toxicant outside the cell. Acquisition of an efflux mechanism facilitates the prevention of the intracellular accumulation of toxic compounds and pumps molecules out of the cell.	*Escherichia coli*	[20] [59]
Decreased influx and increased efflux is not responsible for the K_2_TeO_3_ resistance.	*Escherichia coli*	[65]
Acetate transport system is responsible for uptake of TeO_3_^2−^ and resistance.	*Rhodobacter capsulatus*	[60] [38]
Enzymatic or nonenzymatic reduction of toxic TeO_3_^2−^ (Te^4+^) to insoluble non/less toxic elemental Te as crystals of Te^0^. The reduction TeO_3_^2−^ to Te^0^ can also be carried out by the activity of various types of cytoplasmic, periplasmic and/or membrane-associated reductases.	*Staphylococcus aureus* *Staphylococcus epidermidis* *Pseudomonas aeruginosa* *Escherichia coli* *Rhodobacter sphaeroides* *Bacillus* spp. *Shewanella fridigimarina* *Erythromonas ursincola*	[68] [69] [72] [44] [73] [74] [75]

## Data Availability

Not applicable.
